# Electrochemical affinity biosensors for fast detection of gene-specific methylations with no need for bisulfite and amplification treatments

**DOI:** 10.1038/s41598-018-24902-1

**Published:** 2018-04-23

**Authors:** Eloy Povedano, Eva Vargas, Víctor Ruiz-Valdepeñas Montiel, Rebeca M. Torrente-Rodríguez, María Pedrero, Rodrigo Barderas, Pablo San Segundo-Acosta, Alberto Peláez-García, Marta Mendiola, David Hardisson, Susana Campuzano, José M. Pingarrón

**Affiliations:** 1 Departamento de Química Analítica, Facultad de CC. Químicas, Universidad Complutense de Madrid, E-28040 Madrid, Spain; 2 Unidad Funcional de Investigación de Enfermedades Crónicas, Instituto de Salud Carlos III, 28220 Majadahonda, Madrid Spain; 3 Department of Pathology, Molecular Pathology and Therapeutic Targets Group, Hospital Universitario La Paz IdiPAZ, Madrid, Spain; 4 Molecular Pathology and Therapeutic Targets Group and Molecular Pathology Section, INGEMM, Hospital Universitario La Paz IdiPAZ, Madrid, Spain; 5 Facultad de Medicina, Universidad Autonoma de Madrid, Madrid, Spain

## Abstract

This paper describes two different electrochemical affinity biosensing approaches for the simple, fast and bisulfite and PCR-free quantification of 5-methylated cytosines (5-mC) in DNA using the anti-5-mC antibody as biorecognition element. One of the biosensing approaches used the anti-5-mC as capture bioreceptor and a sandwich type immunoassay, while the other one involved the use of a specific DNA probe and the anti-5-mC as a detector bioreceptor of the captured methylated DNA. Both strategies, named for simplicity in the text as immunosensor and DNA sensor, respectively, were implemented on the surface of magnetic microparticles and the transduction was accomplished by amperometry at screen-printed carbon electrodes by means of the hydrogen peroxide/hydroquinone system. The resulting amperometric biosensors demonstrated reproducibility throughout the entire protocol, sensitive determination with no need for using amplification strategies, and competitiveness with the conventional enzyme-linked immunosorbent assay methodology and the few electrochemical biosensors reported so far in terms of simplicity, sensitivity and assay time. The DNA sensor exhibited higher sensitivity and allowed the detection of the gene-specific methylations conversely to the immunosensor, which detected global DNA methylation. In addition, the DNA sensor demonstrated successful applicability for 1 h-analysis of specific methylation in two relevant tumor suppressor genes in spiked biological fluids and in genomic DNA extracted from human glioblastoma cells.

## Introduction

Growing cancer incidence and mortality worldwide demands the development of new reliable methodologies for the determination of specific cancer biomarkers useful for accurate diagnosis and prognosis of the disease as well as for patient monitoring^1^. It is well known that detection of malignant tumors at an early stage is the key to successful treatment and outcome. Because molecular alterations in neoplastic cells may precede clinically obvious cancer, these changes have emerged as useful targets for such early detection. In addition to DNA sequence aberrations, like point mutations, deletions, rearrangements, or copy number variations, epigenetic modifications, such as DNA methylation, have proven to be an important parameter of neoplastic DNA. In fact, the detection and quantification of epigenetic modifications has become a powerful tool for the detection of both primary and metastatic or recurrent cancer cases and response to treatment^2^.

Unlike genetic mutation, DNA methylation, identified as one of the most frequent molecular phenomenon in human cancers, is a heritable and reversible process that alters gene expression patterns without modifying the DNA sequence^3,4^. Both hypomethylation and hypermethylation states are associated with human cancer and affect different genome regions regarding the cancer type^5^. Hypomethylation, often detected in metastatic tissues and primary tumors increases the expression of oncogenes, activates transcription, and alters genome stability^5,6^. Hypermethylation of CpG-rich genomic regions occurs by altered activity of DNA methyltransferases (DNMTs) and involves the addition of a methyl group to the cytosine ring of those cytosines that precede a guanosine (referred to as CpG dinucleotides) to form 5-methylcytosine (5-mC). This process is considered an early event in the development of cancer^3,7–9^. Aberrant DNA methylation is frequently observed in tumor cells with global hypomethylation and hypermethylation of the CpG islands, which are clusters of CpGs, in the promoter regions of tumor suppressor genes. Indeed, inactivation of tumor suppressor genes, such as the *RASSF1A* and *MGMT*, via hypermethylation of CpG islands located in their regulatory regions, has been shown to be one of the most important mechanisms producing changes in their gene expression and leading to a number of human cancers^4,6,10^. Therefore, the detection of altered DNA methylation patterns in the promoter region of cancer related genes, in DNA derived both from tumor tissues and bodily fluids is considered to be a promising target for early diagnosis of cancers, tumor behavior monitoring, and response to targeted therapy or recognition of recurrence^4,6,11,12^. As a relevant datum to this respect, it was reported that aberrant methylation of the *p16Ink4* and *MGMT* promoters can be detected in DNA from the sputum of patients with squamous cell carcinoma nearly 3 years before clinical diagnosis^13^.

Common methods to detect DNA methylation include fluorescence-based polymerase chain reaction (PCR), sequencing and immuno/affinity reaction biosensing^9^. However, prior to DNA methylation detection using PCR and sequencing methods, DNA samples must be pre-treated either by restriction enzymes digestion or by bisulfite conversion^2,11,14^. During enzymatic digestion, methylation-sensitive restriction enzymes recognized and cleaved unmethylated CpG islands leaving intact the methylated CpG islands. Then the methylation status is assessed in connection with PCR using primers able to amplify only regions containing restriction sites^15^. Despite the usefulness for identifying highly methylated genes, this method is prone to false positive results due to incomplete enzymatic digestion of unmethylated CpG islands^6,15^. Bisulfite conversion changes only unmethylated cytosine bases to uracils via sulphonation, hydrolytic deamination, and alkali desulphonation^16^, and afterwards PCR amplification using primers specific for methylated and unmethylated CpG islands is carried out^15^. While cytosines at unmethylated CpG islands are converted to uracils and bind adenines, methylated CpG islands resist conversion and consequently bind guanines during amplification^15^. However, as with the enzymatic digestion, incomplete bisulfite conversion sometimes causes false positive results. Various locus-based methods have been proposed to establish the methylation status of bisulfite-converted DNA, including methylation-specific PCR (MSP), quantitative real-time MSP (qMSP), and pyrosequencing^6,17^. However, the use of specialized PCR equipment makes these approaches labor-intensive, time-consuming and quite expensive, thus limiting their use in general laboratories. On the other hand, there are several commercially available enzyme-linked immunosorbent assay (ELISA) kits which enable the quick assessment of global DNA methylation profiling using DNA amounts between 100 ng and 2 μg. However, they are typically prone to high variability and, therefore, only suitable for the rough estimation of DNA methylation^18^.

Fluorescence, capillary electrophoresis^19^, colorimetry^20^, surface plasmon resonance^21^ and surface enhanced Raman spectroscopy^22^ have been also employed to detect specific DNA methylation pattern^23^. These methodologies, although effective, tend to be expensive, tedious and DNA and time-consuming for many applications.

In addition, the affinity of the methyl-binding protein (MBD)^24,25^ and the anti-methylcytosine antibody (anti-5-mC)^4,14^ for 5-mC in a double stranded (ds)- or single stranded (ss)-target DNA sequence, respectively, has been exploited to construct a few biosensors for rapid monitoring of DNA methylation^9,26^. However, these biosensors lack nucleic acid amplification, this constraining their sensitivity and applicability for the analysis of real specimens containing low amounts of methylated DNA. Thus, there is still an urgent demand to develop simple, inexpensive and rapid methods for high sensitive monitoring of DNA methylation^11,27^.

Electrochemical techniques have been widely used for sensitive and specific analysis of nucleic acids^28,29^ and also for DNA methylation assays because of their convenience, field portability, intrinsic simplicity, high selectivity, high sensitivity and short analysis time^4,7,23,30–32^. Several PCR independent electrochemical methods for methylated DNA have been reported^4,7,11,23,25,27,33–39^. Although most of the electrochemical affinity biosensors reported so far comply with the required sensitivity^4,24^ using nanomaterials, the variability of the nanomaterials and their bio-functionalization often affect their reproducibility and quantification, especially for real samples^11,40^. Therefore, most of the electrochemical biosensors reported so far were mostly limited to the analysis of synthesized DNA targets, and provided overall pictures of DNA methylation instead of information about distinct patterns in DNA sequence^27^. In addition, their practice in clinical sample analysis has been only scarcely explored^23^.

In this work, aiming at improving the speed, simplicity, sensitivity and usefulness of the biosensors developed for monitoring of DNA methylation, two handy electrochemical biosensing strategies (an immunosensor and a DNA sensor) using neither bisulfite conversion nor nucleic acid amplification are reported to identify the methylation status of cytosine in DNA. The applicability of these strategies has been demonstrated by targeting the promoter methylation status of two normally non-methylated, biologically significant cancer genes, both in spiked biological fluids (*RASSF1A*), and genomic DNA extracted from human glioblastoma cells (*MGMT*).

## Results

Two different electrochemical biosensing strategies for evaluating DNA methylation in a direct and independent PCR mode are described in this work. Specific promoter sequences of *RASSF1A* and *MGMT* genes were selected to verify the suitability of the designed strategies. The RAS association (RalGDS/AF-6) domain family member 1A *RASSF1A* gene is a tumor suppressor gene frequently detected to undergo epigenetic silencing by aberrant hypermethylation of its promoter region in many human solid tumors. It has been reported to have clinical sensitivity of 67–75% for stage IV breast cancer^2,41,42^. On the other hand, methylation of the O6-methylguanine-DNA methyltransferase (*MGMT*) of the *MGMT* promoter of malignant glioma appears to be a useful predictor of the responsiveness to alkylating agents that reverse epigenetic alterations. Numerous studies have demonstrated that patients with silencing *MGMT* respond better to therapy^13,43^.

**Figure 1:**
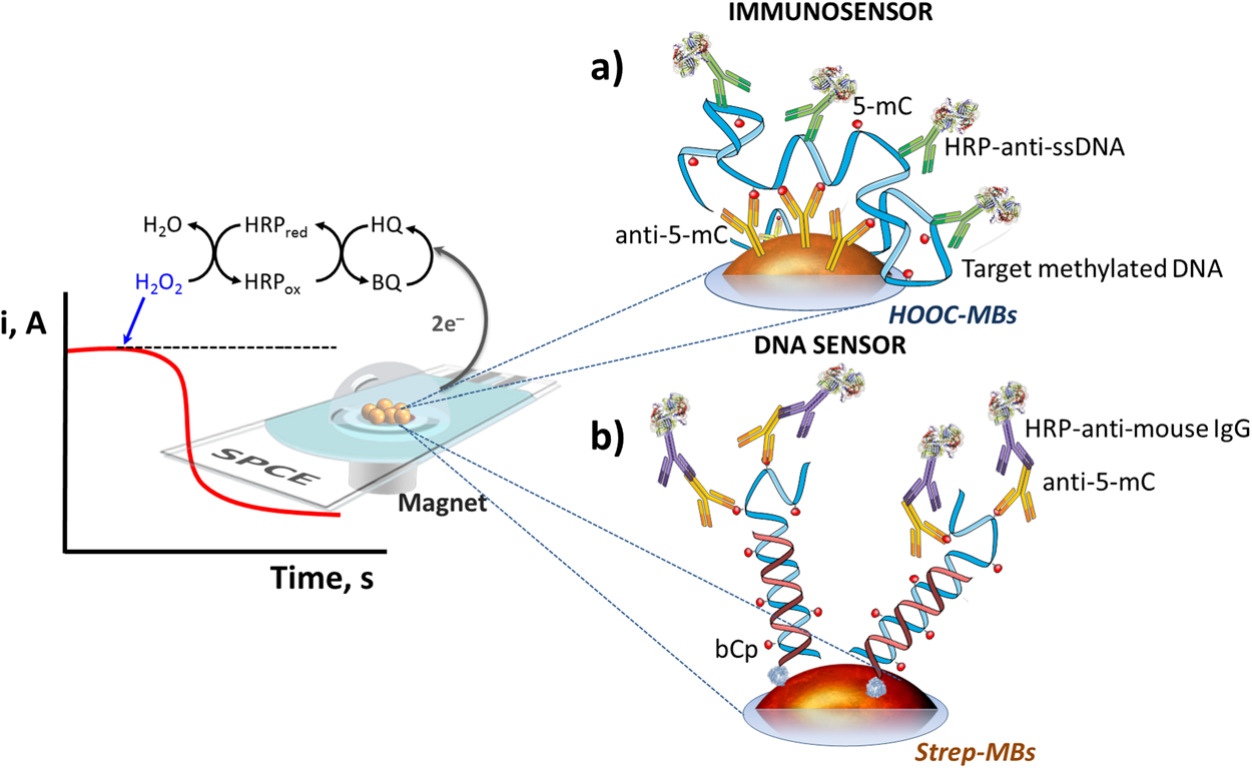
Schematic display of the biosensors’ fundamentals. Schematic display of the immunosensor (**a**) and the DNA sensor (**b**) developed for the determination of 5-mC methylation and the amperometric detection using the H_2_O_2_/HQ system at the SPCE.

The first strategy (Fig. 1a) is an immunosensor involving the use of two different antibodies. The one immobilized on the surface of carboxylic acid-modified magnetic beads (HOOC-MBs), is specific to 5-methylcytosine (anti-5-mC) and, therefore, capable of capturing any ss-DNA sequence bearing this type of methylation. A second antibody conjugated with peroxidase (HRP-anti-ssDNA), able to recognize any ss-DNA, was used as detector antibody.

The second method (Fig. 1b) is a DNA sensor involving immobilization of a biotinylated DNA capture probe, specific to the methylated sequence to be detected, on the surface of Streptavidin-modified MBs (Strep-MBs). Methylation in the captured target DNA was recognized by means of the specific anti-5-mC tagged with a secondary HRP-conjugated antibody (HRP-anti-mouse IgG). In both strategies, amperometric determination was carried out using the hydrogen peroxide/hydroquinone (H_2_O_2_/HQ) system after magnetic capture of the modified MBs on the surface of a screen-printed carbon electrode (SPCE), by measuring the cathodic current generated by the enzymatic reduction of H_2_O_2_ mediated by HQ, this current being proportional to the concentration of methylated DNA in the sample.

### Optimization of the experimental conditions

Because methylation frequently is present in a little subset of cells in a clinical specimen, the sensitivity is critical to design an analytical method for monitoring the methylation status of the target gene^11,44^. Therefore, seeking for a high sensitivity and for simple and short assay protocols, the relevant experimental variables involved in the biosensors preparation were optimized. The taken selection criterion was the largest ratio between the current values measured at a potential value of −0.20 V (*vs*. the Ag pseudo-reference electrode) using the H_2_O_2_/HQ system, in the absence (B) and in the presence of 5.0 nM of synthetic target *RASSF1A* (S). The evaluated variables, the tested ranges and the values selected for further work are summarized in Table 1. Other experimental variables not included in the Table, such as the MBs suspension volume^45,46^ and the potential applied for the amperometric response^47^ were optimized in previous works.

**Table 1:** Optimization of the experimental variables affecting the performance of the amperometric biosensors developed for quantification of DNA methylation.

Biosensor	Variable	Evaluated range	Selected value
Immunosensor	[anti-5-mC], µg mL^−1^	0.4–10	4
	anti-5-mC incubation time, min	15–90	60
	HRP-anti-ssDNA dilution	1/50–1/500	1/100
	HRP-anti-ssDNA incubation time, min	15–60	15
	Target DNA incubation time, min	15–60	30
DNA sensor	[bCp], µM	0.01–0.5	0.1
	bCp incubation time, min	15–60	15
	Number of steps	1–3	2
	Target DNA incubation time, min	15–60	30
	[anti-5-mC], µg mL^−1^	0.25–2.0	0.5
	[HRP-anti-mouse IgG], µg mL^−1^	0.125–2.0	1.0
	anti-5-mC + HRP-anti-mouse IgG mixture incubation time, min	15–120	30

**Figure 2:**
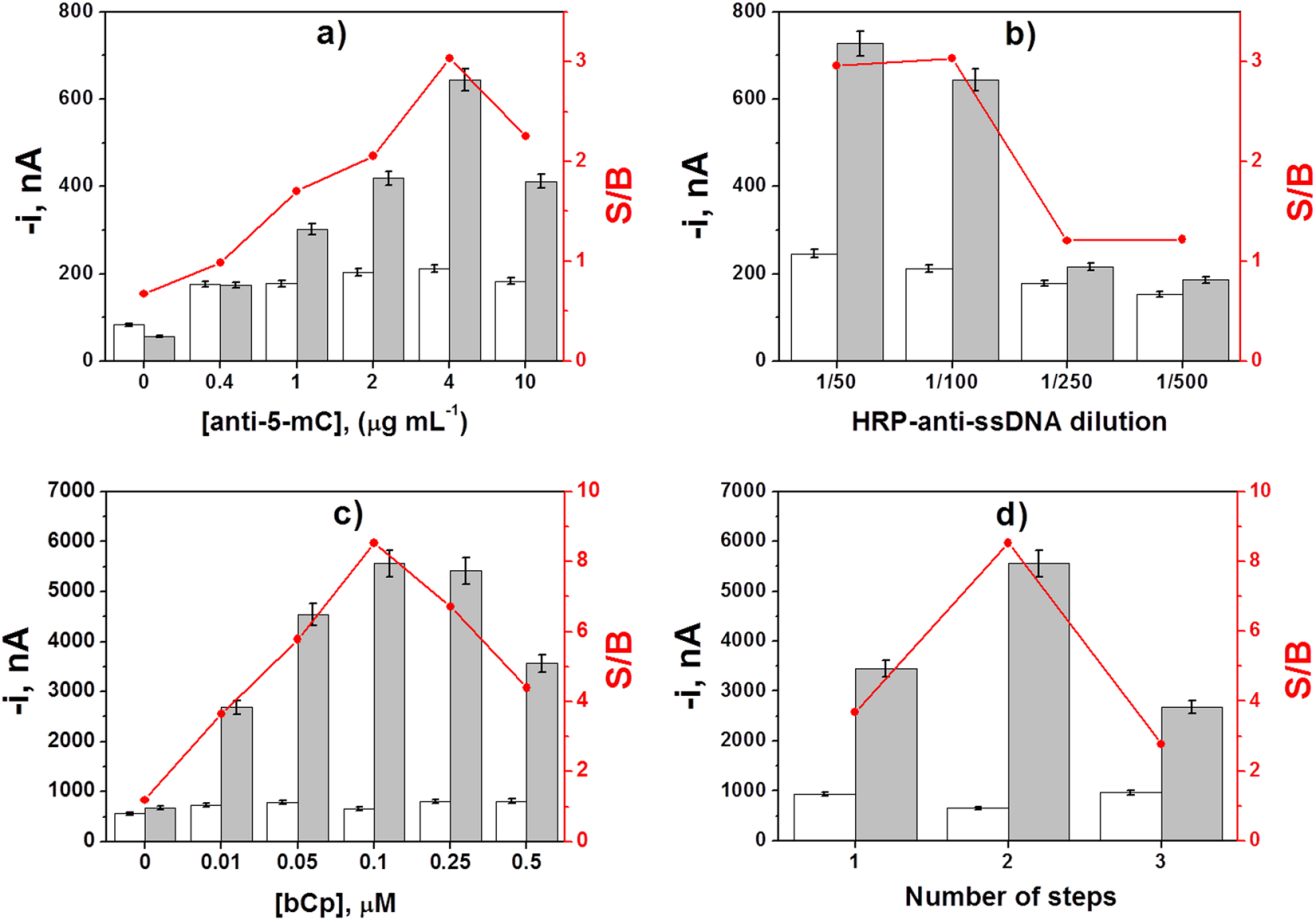
Optimization of experimental variables. Influence of the capture (**a**) and detector (**b**) antibodies concentration used in the immunosensor fabrication, and the bCp concentration (**c**) and the number of steps (**d**) used in the preparation of the DNA sensor, on the amperometric responses measured with the developed biosensors for 0.0 (white bars) and 5.0 nM of synthetic target *RASSF1A* (grey bars) and the corresponding S/B ratio values (in red). Error bars estimated as triple of the standard deviation of three replicates. Data presented in (**a**–**c**) have been obtained using the optimal protocol described in MBs modification subsection involving 2 steps.

Illustrative examples of these optimization studies, such as the capture and detector antibodies loadings in the immunosensor and the biotinylated capture probe (bCp) concentration and number of steps for the DNA sensor, are displayed in Fig. 2a–d. Figure 2a and b show as the S/B ratio increased with the concentration of the capture and detector antibodies up to a certain concentration and then decreased significantly due to the sterically hindered antigen binding when high loadings of antibodies are immobilized^48^. The somewhat higher signal obtained in the absence than in the presence of target methylated DNA without anti-5-mC (Fig. 2a) may be attributed to a slightly higher non-specific adsorption of HRP-anti-ssDNA on HOOC-MBs in this case.

Regarding the optimization of the bCp concentration in the DNA sensor (Fig. 2c), while no significant differences were apparent between the B signals, the S signals increased significantly with the bCp concentration up to 0.1 μM, then decreasing for larger concentrations, which is most likely due to the restricted hybridization efficiency when large amounts of bCp are immobilized^49,50^. Results achieved in the optimization of all the other experimental variables are shown in Figure S1 (in the Supplementary Information).

Looking for a protocol simplification with reduced assay time, the influence of the number of steps used in the preparation of the DNA sensor was also investigated. All the evaluated protocols started after preparation of bCp-MBs and involved 30 min-incubation steps. The tested protocols consisted of: (a) incubation of bCp-MBs with a mixture solution containing the target DNA, the anti-5-mC and the HRP-anti-mouse IgG (1 step); (b) a former incubation with the target DNA solution and a second one with an anti-5-mC and the HRP-anti-mouse IgG mixture solution (2 steps); (c) independent and successive incubations with target DNA, anti-5-mC and HRP-anti-mouse IgG solutions (3 steps). Figure 2d shows as the working protocol involving two incubation steps provided the largest S/B current ratio. This protocol, additionally, allows reducing largely the total assay time. The observed results could be attributed to a less efficient hybridization of the target DNA labelled with both antibodies onto the bCp-MBs (1 step), and to a better recognition of the anti-5-mC by the HRP-anti-mouse IgG when both antibodies are free in solution. Consequently, a 2-step protocol involving the former hybridization of the target DNA onto the bCp-MBs and further labeling of the captured methylated DNA by incubation in an anti-5-mC and HRP-anti-mouse IgG mixture solution was selected for further work.

### Analytical characteristics

**Figure 3:**
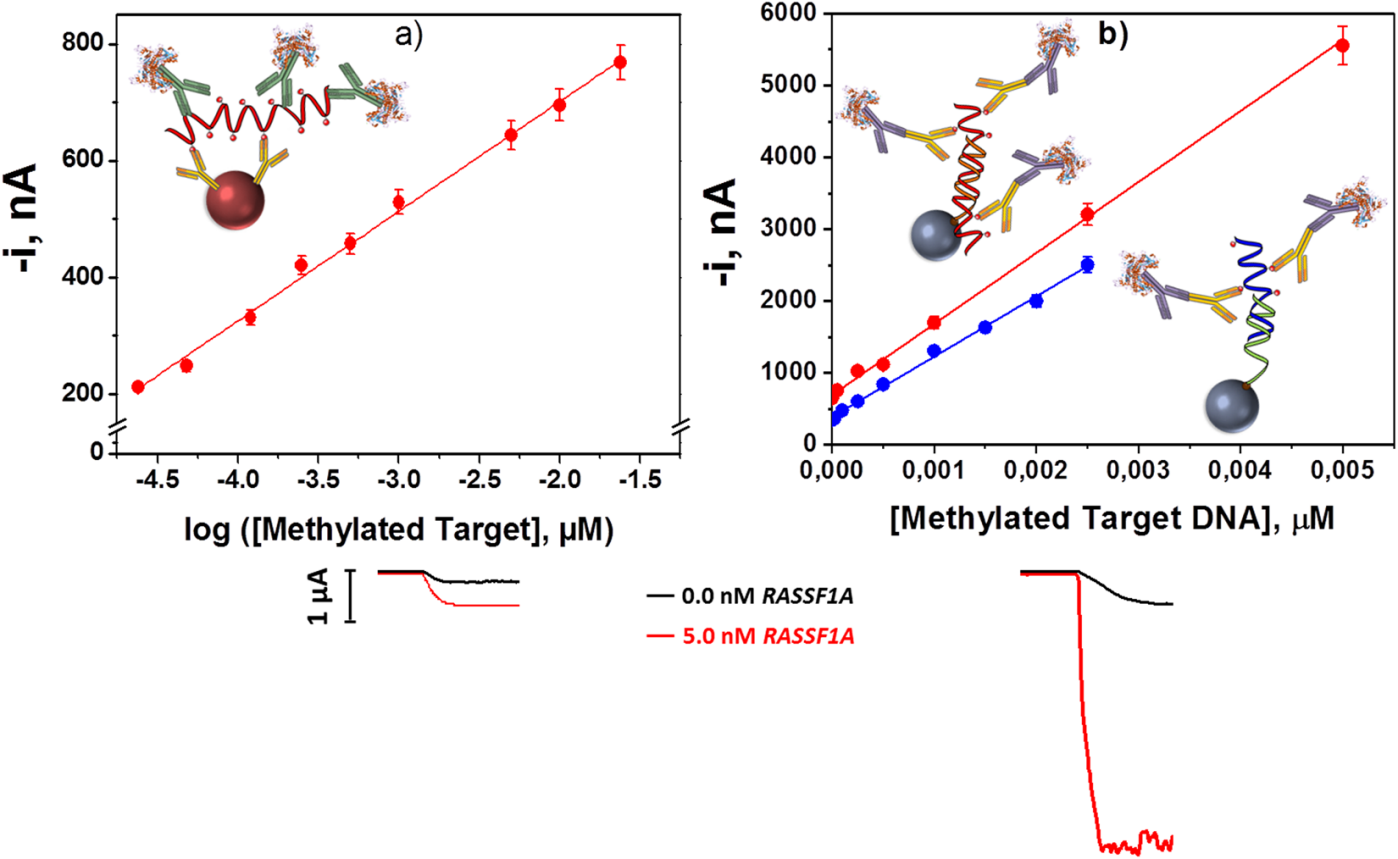
Standards calibration plots. Calibration plots constructed with the immunosensor (**a**) and the DNA (**b**) biosensors for the methylated synthetic sequences of the promoter regions of *RASSF1A* () and *MGMT* () genes. Amperometric responses obtained with the immunosensor (left) and the DNA sensor (right) in the absence and in the presence of 5.0 nM of the target *RASSF1A*. Error bars estimated as triple of the standard deviation of three replicates.

The calibration plots and the analytical characteristics obtained with the two developed biosensing strategies for the methylated synthetic sequences of the promoter regions of the selected genes are displayed in Fig. 3 and summarized in Table 2. The limit of detection (LOD) values achieved allowed the detection of 0.3–1.0 fmol of methylated target gene’s promoter. The reproducibility of the amperometric responses obtained with different biosensors prepared in the same manner was evaluated by comparing the current values measured for 0.5 and 1.0 nM of synthetic target for the immuno- and DNA sensor, respectively. As it can be observed in Table 2, the relative standard deviation (RSD) values demonstrated great reproducibility in both biosensors preparation protocols as well as in the amperometric transduction.

**Table 2:** Analytical characteristics obtained with the biosensors developed for the determination of the synthetic methylated target DNA sequences of the promoter region of the *RASSF1A* and *MGMT* genes.

Parameter	Immunosensor	DNA sensor	
Linear dependence	i_c_ *vs*. log [*RASSF1A*]	i_c_ *vs*. [*RASSF1A*]	i_c_ *vs*. [*MGMT*]
r	0.997	0.999	0.999
Slope	(188 ± 3) nA	(98 ± 1) × 10^4^ nA µM^−1^	(84 ± 1) × 10^4^ nA µM^−1^
Intercept, nA	(1,077 ± 11) nA	(710 ± 20) nA	(390 ± 16) nA
Linear range, pM	23–24,000	139–5,000	87–2,500
LOD, pM	6.8 (0.3 fmol, 4.8 pg)	42 (1.0 fmol, 16.0 pg)	26 (0.6 fmol, 9.7 pg)
RSD, %*	3.9 (n = 10, 0.5 nM)	4.8 (n = 7, 1.0 nM)	4.3 (n = 7, 1.0 nM)
Assay time, min	45	60	60

^*^indicated in parentheses the concentration at which RSD has been calculated.

When we compared the sensitivity achieved with the developed immunosensor with a commercial ELISA methodology for the same methylated DNA standard (*E*. *coli* genomic DNA with 346,670 methylated cytosines), we observed that the immunosensor provided a 2,500 times lower LOD (0.004 *vs*. 10 ng) in a 4-times shorter assay time (45 min *vs*. 3 h). Also, the LOD achieved with the immunosensor is 4.7-times lower (6.8 *vs*. 32 pM) than that reported for an electrochemical immunosensor for 5-hmC methylation (determination of 5-hydroxymethyl-2′-deoxycytidine-5′-triphosphate)^36^.

It is important to remark that both developed methodologies allowed methylated DNA to be determined at picomolar level in about 1 h, with no need for bisulfite and amplification pretreatments and excellent reproducibility throughout the entire protocols. However, the DNA sensor exhibits better sensitivity (see comparative amperograms in (Fig. 3b). Moreover, the existence of 5 methylated cytosines in the part of the sequence that remains unhybridized (the only ones recognized by the anti-5-mC)^14^ in the synthetic target *RASSF1A* compared with the 4 in the *MGMT*, apart from the longest length of the hybrid fragment, explains the slightly higher sensitivity achieved for the determination of the target *RASSF1A*.

Interestingly, a comparison between the electrochemical methods reported so far for the determination of different types of targets (synthetic oligonucleotides, free DNA bases, PCR products and genomic DNA), demonstrates that the LODs achieved with the DNA sensor for the synthetic short target methylated DNAs are much lower than those reported with PCR amplification (25 pg)^11^, as well as for PCR-free methods using direct oxidation of DNA bases of short methylated oligonucleotides (0.11 μM)^35^ and free un-methylated C (0.6 μM)^38^, digestion by a restriction enzyme of a synthetic target DNA (10 nM)^27^, bisulfite conversion of synthetic target DNAs (18 pM)^34^ and PCR products (0.5 nM)^37^, and a paired-end tagging and amplification electrochemical strategy for methylated genomic DNA (40 pg)^23^. It is important to note also that the most sensitive strategies among these required assay times between 1.5 and 24.5 h^11,23,34^ compared to the 1 h of the DNA sensors developed in this work.

It is worth to mention also that although the LODs achieved with the developed biosensors are higher than those claimed for other two reported affinity electrochemical biosensors for synthetic target methylated DNAs determination, 2 fM^4^ and 35 fM^24^, the preparation of these biosensors required multiple reagents and complex and time-consuming working protocols that included amplification strategies involving nanomaterials. Moreover, in both cases the assay time is 3–5 times longer than that needed with the developed biosensors (2 h 40 min^4^ and 4 h 50 min)^24^. It is important to highlight also that, apart from the shorter assay time and the inherent simplicity of the biosensors construction, these allow an accurate and straightforward determination of the synthetic target methylated DNA in spiked biological samples without previous extraction or amplification of the genetic material, as it will be shown in further on.

In addition, the storage stability of the anti-5-mC-MBs and the bCp-MBs employed for the preparation of the immuno- and DNA sensors, respectively, was evaluated by storing the modified MBs at 4 °C in microcentrifuge tubes containing 50 μL of filtered phosphate-buffered saline (PBS) or Binding and Washing buffer (B&W), respectively. Subsequently, they were used to prepare biosensors on different working days and to measure the amperometric responses for 0.0 and 0.5 nM of target *RASSF1A* or 1.0 nM of target *MGMT* solutions, respectively. No significant decrease in the measured S/B ratio was observed during at least 35 days in both cases (no longer times were assayed), suggesting the possibility of preparing the conjugated MBs in advance and storing them under the above-described conditions, until the biosensor preparation is required.

### Selectivity

**Figure 4:**
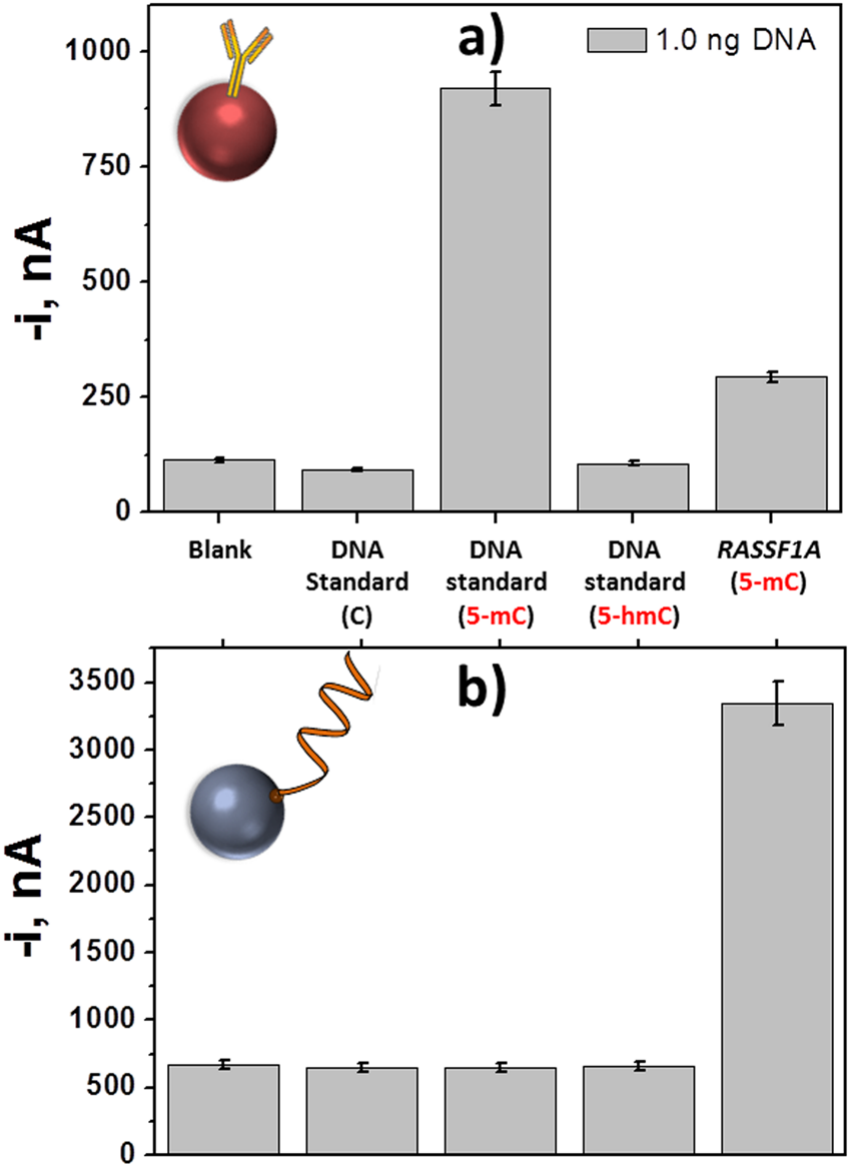
Selectivity of biosensors. Comparison of the amperometric responses obtained with the inmunosensor (**a**) and the DNA sensor for *RASSF1A* promoter region (**b**) in the absence of DNA (blank) and in the presence of 1 ng of unmethylated and methylated with 5-mC and 5-hmC DNA standards and the synthetic methylated target DNA sequence of the *RASSF1A* and *MGMT* promoters region. Error bars estimated as triple of the standard deviation of three replicates.

The selectivity of both biosensors towards 5-mC was evaluated by comparing the amperometric responses they provided for 1.0 ng of denatured synthetic DNA standards containing unmodified cytosines (C), 5-mC or 5-hmC. Results shown in Fig. 4 demonstrate the specificity of the developed methodologies to detect only 5-mC sequences. In addition, as predicted, the immunosensor detected the presence of any oligonucleotide with 5-mC methylation without any sequence selectivity. Accordingly, this sensor was able to quantify the total amount of 5-mCs in the analyzed DNA. Conversely, the DNA sensor only recognized the methylated sequence complementary to the bCp immobilized on the functionalized MBs and thus it can be used to simultaneously detect the presence of 5-mCs and their position in the DNA sequence.

### Detection of 5-mC DNA methylation in biofluids and cells

The evaluation of the usefulness of the developed methodologies was restricted to the DNA sensor because of its higher sensitivity and suitability for quantification of gene-specific methylation as compared with the immunosensor. Such evaluation was accomplished in biological fluids supplemented with synthetic methylated target *RASSF1A*.

**Figure 5:**
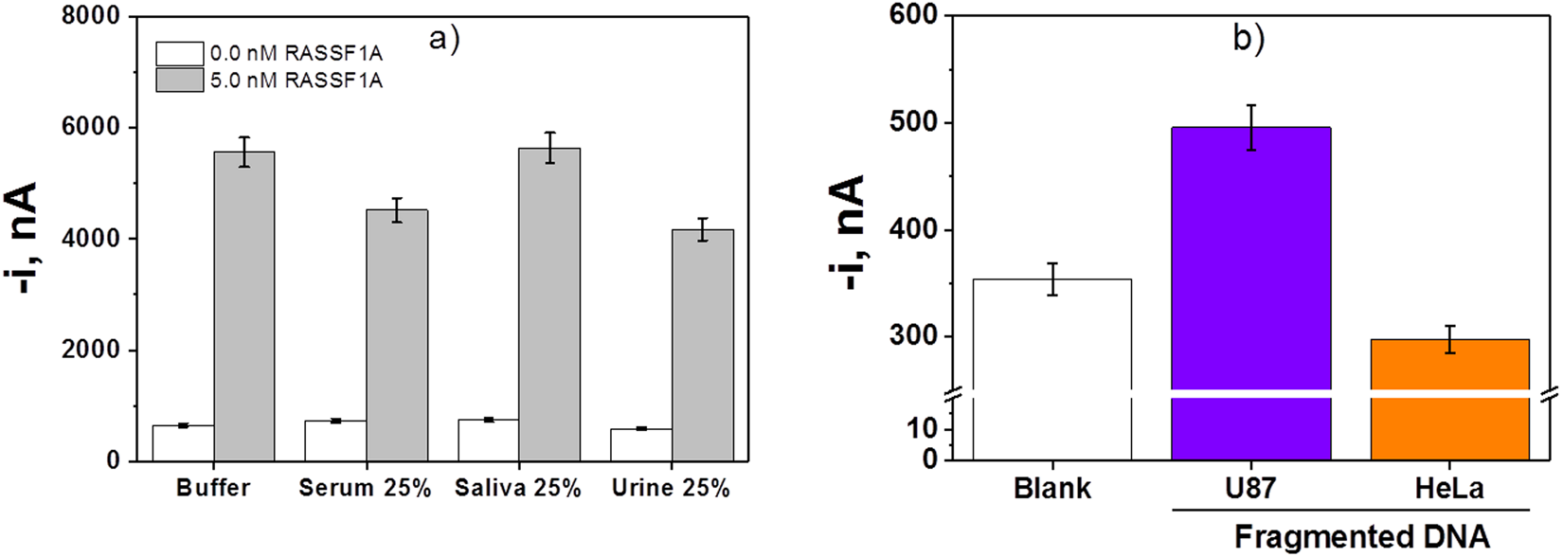
Analysis of spiked biological samples. Comparison of the amperometric responses provided by the DNA sensor in the absence and in the presence of 5.0 nM of the synthetic target *RASSF1A* prepared in different media. Error bars estimated as triple of the standard deviation of three replicates (**a**). Amperometric responses measured with the DNA biosensor in the absence of synthetic target *MGMT* and in the presence of 100 ng of fragmented genomic DNA extracted from U87 cells and HeLa cells (**b**).

The comparison of the amperometric responses obtained with the DNA sensor in the absence and in the presence of 5.0 nM (125 fmol) of the synthetic target *RASSF1A* prepared in buffered solution and in the different biological fluids tested 25%-diluted with 5-mC-ELISA buffer is shown in (Fig. 5a). The slope values corresponding to calibration plots prepared between 139 and 5,000 pM of the synthetic target *RASSF1A* in each media are summarized in Table 3. The comparison of the obtained slope values revealed the existence of matrix effects in serum and urine samples and, therefore, in these biological samples the determination should be carried out by interpolation in the representative calibration plots constructed in the 25%-diluted biological fluids instead of the calibration constructed for synthetic target in buffer (case of saliva samples). The results obtained in the recovery studies performed in these biological samples spiked with 2.5 nM of the synthetic target *RASSF1A* for a confidence level of 0.95 (summarized in Table 3) outline the reliability of the approach to determine a low concentration of the synthetic target in biological fluids just after a simple dilution and without prior DNA extraction and preconcentration. These results are considered particularly relevant taking into account that the commercial ELISA methods, as well as most of the biosensors described so far for methylation determination, have proved to be suitable only for the analysis of previously extracted DNA, but not directly in biological samples, and after applying bisulfite and/or amplification treatments.

**Table 3:** Slope values obtained with the DNA sensor for the calibration plots obtained for synthetic target *RASSF1A* prepared in buffer and in different biological samples and results obtained in the recovery studies performed with the developed DNA sensor in samples spiked with 2.5 nM of the synthetic target *RASSF1A*.

Medium	Slope, nA µM^−1^	Recovery_(n=3)_, %
Buffer	(98 ± 1) × 10^4^	101 ± 4
Serum 25%	(76 ± 7) × 10^4^	97 ± 5
Saliva 25%	(98 ± 14) × 10^4^	95 ± 6
Urine 25%	(72 ± 6) × 10^4^	94 ± 7

It is important to note that most of the work reported in the literature only provides semi-quantitative data or comparative percentages of methylation in healthy and cancer patients. Therefore, it is extremely difficult to find any reference indicating the absolute value of the methylated target DNA concentration in liquid biopsies samples. Some indication about the concentration of circulating hypermethylated *RASSF1A* in serum from breast cancer patients, between 1 and 200 ng mL^−1^, is given by Kristiansen *et al*.^42^, while the best cut-off for circulating serum *RASSF1A* to differentiate the hepatocellular carcinoma is 13 pM according to Mansour *et al*.^51^.

Despite the interesting results achieved in spiked serum, saliva and urine, it is important to mention that in most clinical applications DNA methylation detection at specific gene positions is much more relevant than quantification of DNA methylation. Therefore, in order to check the clinical applicability of the developed DNA sensor, the implemented methodology was applied to analyze the endogenous *MGMT* status in the reference cell line U87. Figure 5b shows that a larger amperometric response was measured when 100 ng genomic DNA extracted from these cells were analyzed, in comparison with the currents measured for both in the absence of target DNA and genomic DNA extracted from HeLa cells (non-methylated *MGMT* gene promoter cells used as control)^52^, which is consistent with the reported specific hypermethylation of the *MGMT* gene of U87 cell line^53,54^.

All these results confirmed that the developed electrochemical DNA sensor exhibits suitable sensitivity and specificity for the determination in just 1 h of gene-specific methylations directly in biological fluids without previous DNA extraction and pretreatments (bisulfite and/or amplification), and in genomic DNA extracted from cells.

## Discussion

This paper describes the development of two electrochemical biosensing strategies free of bisulfite and/or amplification pretreatments for the simple, sensitive and quick detection of DNA methylation using functionalized MBs, the anti-5-mC as affinity bioreceptor and amperometric detection at SPCEs using the H_2_O_2_/HQ system. While the immunosensor uses the anti-5-mC immobilized onto HOOC-MBs as capture bioreceptor, the DNA sensor employs the anti-5-mC as detector bioreceptor to label the methylated DNA previously captured by a complementary DNA capture probe immobilized on the surface of Strep-MBs. Both sensors types imply simple and reproducible working protocols free of the complex and time consuming procedures required by the conventional methodologies. However, the DNA sensor exhibits a higher sensitivity and a clear discrimination only for the methylated sequence complementary to the immobilized capture probe. The obtained results demonstrated the suitability of the DNA sensor for detection of the gene-specific methylation without PCR amplification, bisulfite or labeling processes, directly in genomic DNA extracted from cells and in liquid biopsies without previous DNA extraction or amplification. In comparison with conventional ELISA available approaches, the developed sensors can greatly benefit research on DNA methylation in terms of convenience, low-cost, ease of operation and suitability for universal analysis of any DNA sequence. These sensors offer also very interesting features over other electrochemical biosensors described to date in terms of sensitivity and assay time. Moreover, the fact that they do not require complicated nanomaterials preparation, conventional methylated DNA pretreatments or sophisticated instruments for signal readout makes them economical and compatible with portable and low-cost devices to perform studies of DNA methylation-related functional genomics and epigenomics in different settings or at the *point-of-need*. These attractive capabilities can make these biosensing strategies to serve as a basis for developing easy and affordable tests in an easy-to-use format, not too technically demanding and requiring equipment readily available at most academic institutions for cancer suspects as well as for management and better outcome of cancer patients, complementing current and future pathological and molecular assessments.

## Methods

### Apparatus and electrodes

Amperometric measurements were performed with a CH Instruments (Austin, TX) model 812B potentiostat controlled by software CHI812B. SPCEs (DRP-110), consisting of a 4-mm diameter carbon working electrode, a carbon counter electrode and an Ag pseudo-reference electrode were used as electrochemical transducers, and the specific cable connector (DRP-CAC), which acted as interface between the SPCEs and the potentiostat, were purchased from DropSens (Spain). All the electrochemical measurements were performed at room temperature. A neodymium magnet (AIMAN GZ) embedded in a homemade Teflon casing was used for the reproducible magnetic capture of the modified-MBs on the surface of SPCEs.

A Bunsen AGT-9 Vortex for homogenization of the solutions, a Raypa steam sterilizer, a biological safety cabinet Telstar Biostar, a thermocycler (SensoQuest LabCycler, Progen Scientific Ltd.), an incubator shaker Optic Ivymen® System (Comecta S. A, Sharlab) and a magnetic particle concentrator DynaMag™-2 (123.21D, Invitrogen Dynal AS) were also employed.

### Reagents and Solutions

All reagents used were of the highest available analytical grade. Strep-MBs (2.8 µm Ø, 10 mg mL^−1^, Dynabeads M-280 Streptavidin, 11206D) and HOOC-MBs (2.7 μm Ø, 10 mg mL^−1^, Dynabeads M-270 carboxylic acid, Cat. No: 14305D) were purchased from Thermo Fisher Scientific. NaCl, KCl, NaH_2_PO_4_, Na_2_HPO_4_ and Tris–HCl were purchased from Scharlab. 2-(N-morpholino)ethanesulfonic acid (MES) was purchased from Gerbu. N-(3-dimethylaminopropyl)-N′-ethylcarbodiimide (EDC), N-hydroxysulfosuccinimide (sulfo-NHS) were purchased from Fluorochem. Ethanolamine, HQ and H_2_O_2_ (30%, w/v) were purchased from Sigma-Aldrich.

Mouse anti-5-methylcytosine monoclonal antibody (anti-5-mC), anti-DNA antibody conjugated with HRP (HRP-anti-ssDNA) and 897 bp linear dsDNA standards containing 201 cytosines either unmodified (C DNA standard), 5-methylcytosine (5-mC DNA standard) or 5-hydroxymethylcytosine (5-hmC DNA standard) from DNA Standard Set (Cat. No. D5405) were purchased from Zymo Research. An HRP-labeled anti-mouse IgG from Abcam was also used.

Human sera from clotted whole blood (in lyophilized powder) were purchased from Sigma-Aldrich. Saliva and urine samples were collected from healthy volunteers. A Salivette® collection device (Sarstedt) was used for collecting the saliva samples. Fragmented HeLa genomic DNA used as control in the cells experiments was a component of the EpiMark Methylated DNA Enrichment Kit (New England Biolabs, Inc.).

The following buffer solutions, prepared with Milli-Q water (18 MΩ cm at 25 °C) and sterilized after their preparation, were used: PBS consisting of 0.01 M phosphate buffer solution containing 137 mM NaCl and 2.7 mM KCl, pH 7.5, 0.1 M Tris–HCl, pH 7.2, 0.1 M phosphate buffer, pH 8.0; 0.025 M MES buffer, pH 5.0, B&W buffer consisting of 10 mM Tris–HCl solution containing 1 mM EDTA and 2 M NaCl, pH 7.5, Tris–EDTA buffer (TE) consisting of 0.01 M Tris–HCl solution containing 1 mM EDTA, pH 8.0 and 0.05 M phosphate buffer, pH 6.0. Commercial 5-mC ELISA Buffer (Cat. No.: D5325-2-250) and 10 × ELISA Buffer (Cat. No.: D5425-2-30) from Zymo Research were also used.

All DNA synthetic oligonucleotides used were purchased from Integrated DNA Technologies and their sequences are summarized in Table 4. All of them were reconstituted upon reception in TE buffer to a final concentration of 100 μM, divided into small aliquots and stored at −80 °C.

**Table 4:** Oligonucleotides used in this work.

Name	Sequence 5′ → 3′
bCp-RASSF1A*	TCGCGCAACCGTGCGAGGTCGGCC–Biotin
Target *RASSF1A*	CC(M)GGC(M)GTGGGCC(M)GACCTC(M)GCAC(M)GGTTGC(M)GC(M)GAC(M)GC(M)GTAGC(M)GC
bCp-MGMT*	Biotin-CACCAAGTCGCAAACGGTGCGCAC
Target *MGMT*	GTCCC(M)GAC(M)GCCC(M)GCAGGTCCTC(M)GCGGTGCGCACCGTT

C(M): 5-methylcytosine (5-mC).

*The positions of biotin in both Cps were selected to place the high number of 5-mCs in the synthetic targets farther away from the surface of MBs to make there more accessible for anti-5-mC recognition.

It is worth to mention at this point that the probe and target methylated DNA strands were designed according to the specific promoter sequence of *RASSF1A*^4^ and *MGMT*^55^ genes, and that methylated and unmethylated target DNAs have similar hybridization capacity to probe oligonucleotides^27^.

### MBs modification

MBs modification was different depending on the type of biosensor prepared (Fig. 1). The following protocols were used:

- Immunosensor (Fig. 1a): A 3-μL aliquot of the HOOC-MBs commercial suspension was placed into a 1.5 mL Eppendorf tube and washed twice with 50 μL of MES buffer for 10 min (25 °C, 950 rpm). Thereafter, carboxyl groups of the magnetic microcarriers were activated by incubation in 25 μL of a freshly EDC/sulfo-NHS solution (50 mg mL^−1^ each, in MES buffer, pH 5.0) for 35 min (25 °C, 950 rpm) and, after two washing steps with MES buffer, the microbeads were incubated in 100 μL of an anti-5-mC solution (4 µg mL^−1^ in 0.025 M MES buffer, pH 5.0) for 60 min (37 °C, 950 rpm). Subsequently, the anti-5-mC-MBs were washed twice with 50 μL of MES buffer (0.025 M, pH 5.0) and incubated with 25 μL of 1 M ethanolamine solution (prepared in 0.1 M phosphate buffer, pH 8.0) for 60 min (25 °C, 950 rpm). After this blocking step, three washings were carried out with 50 µL Tris–HCl (0.1 M, pH 7.2), the first one, and with 1 × ELISA buffer (prepared by dilution from the 10 × ELISA buffer), the following two. Subsequently, the anti-5-mC-MBs were incubated with 48 μL of the target DNA solution (RASSF1A) for 30 min (37 °C, 950 rpm). Two washings were performed with 50 μL 1 × ELISA buffer and the target DNA/anti-5-mC-MBs were incubated with 25 μL of an HRP-anti-ssDNA solution (dil. 1/100 in 1 × ELISA buffer) for 15 min (25 °C, 950 rpm). Finally, two washings were carried out with 50 μL of 1 × ELISA buffer and the modified particles were re-suspended in 50 μL of phosphate buffer solution (0.05 M, pH 6.0) to carry out the amperometric measurement.
- DNA sensor (Fig. 1b): 5 μL of the Strep-MBs commercial suspension were deposited in a 1.5 mL Eppendorf tube. After performing two washing steps with 50 µL of B&W buffer solution (pH 7.5), the Strep-MBs were re-suspended and incubated for 15 min (37 °C, 950 rpm) in 25 µL of 0.1 µM of the corresponding b-DNACp solution (bCp-*RASSF1A* or bCp-*MGMT*) prepared in B&W (pH 7.5). Thereafter, the b-DNACp-MBs were washed twice with 50 μL of 5-mC-ELISA buffer and incubated in 25 µL of the synthetic target DNA (*RASSF1A* or *MGMT*) solution (prepared in 5-mC-ELISA buffer) for 30 min (37 °C, 950 rpm). Subsequently, two additional washings were carried out with 50 µL of the 5-mC-ELISA buffer, and the target/b-DNACp-MBs were incubated for 30 min (37 °C, 950 rpm) with 100 μL of an anti-5-mC (0.5 μg mL^−1^) and anti-IgG-HRP (1.0 μg mL^−1^) mixture solution in 5-mC-ELISA buffer. After two final washings with 50 μL of 5-mC-ELISA buffer, the modified particles were resuspended in 50 µL of phosphate buffer solution (0.05 M, pH 6.0) to perform the amperometric detection.

### Amperometric detection

In both types of sensors, the modified MBs were magnetically and reproducibly captured on the working carbon electrode surface by pipetting 50 μL of the modified MBs suspension onto the SPCE upon allocating it on a homemade Teflon casing with an encapsulated neodymium magnet. Then, the SPCE/magnet holding block ensemble was immersed into an electrochemical cell containing 10 mL of 0.05 M phosphate buffer of pH 6.0 and 1.0 mM HQ (prepared just before performing the electrochemical measurement). Amperometric measurements in stirred solutions were made at −0.20 V *vs*. Ag pseudo-reference electrode. Once the background current was stabilized, 50 μL of a 0.1 M H_2_O_2_ solution were added and the generated current recorded until the steady-state current was reached (∼100 s). The amperometric measurements given through the whole manuscript corresponded to the difference between the steady-state and the background currents and are the average of at least three replicates (confidence intervals calculated for α = 0.05). The LOD values were estimated according to the 3 × s_b_/m criteria, s_b_ being the standard deviation (n = 10) for measurements made in the absence of target DNA and m the slope value of the corresponding calibration plot.

### Analysis in cells and spiked biological fluids

The applicability of the developed methodology in biological fluids was evaluated by comparing the sensitivity achieved in buffered solutions with those obtained in biological samples (human saliva, serum and urine) spiked with increasing concentrations of the methylated synthetic sequence of the *RASSF1A* promoter region and performing recovery studies in these complex matrices.

In addition, U87 cells were grown at 37 °C in a humidified atmosphere containing 5% CO_2_ and maintained in high-glucose DMEM (Dulbecco’s modified Eagle’s medium), supplemented with fetal bovine serum (10%), penicillin (100 U mL^−1^), streptomycin (100 μg mL^−1^), and L-glutamine (2.5 mM) (GIBCO-Invitrogen, Carlsbad, CA, USA).

Genomic DNA was isolated from these cells with QIAamp DNA FFPE Tissue Kit (Valencia, CA, USA) according to the manufacturer’s instructions with minor modifications. DNA concentrations and quality were measured using a Nanodrop 1000A spectrophotometer (Wilmington, DE, USA), obtaining ratio values confirming pure DNA in all cases. Due to DNA from tissue biopsies is fragmented when isolated from FFPE, genomic DNA from U87 was sonicated 3 cycles of 10 s at 30% amplitude for fragmentation.

The analysis of genomic DNA extracted from cells and the 897 bp linear dsDNA standards involved their previous denaturation by heating at 97 °C for 5 min in a thermocycler and transferring immediately to ice for 10 min just before making the determination with the biosensor using a similar protocol to that followed with the synthetic target DNAs.

This study and all the experimental protocols used were performed according to the guidelines and regulations and approved by the University Complutense of Madrid. It is worth to mention that since the samples analyzed were commercial serum samples and urine and saliva collected from one of the authors of this paper (V. Ruiz-Valdepeñas Montiel) no other written informed consents were required.

## Electronic supplementary material

Supplementary Information
