# Supplementary material for: Electrochemical affinity biosensors for fast detection of gene-specific methylations with no need for bisulfite and amplification treatments
Source: Sci Rep. 2018 Apr 23;8:6418. doi: 10.1038/s41598-018-24902-1 (PMC5913137; doi:10.1038/s41598-018-24902-1)
Supplement: Supplementary file 1 — Supplementary Information [file 41598_2018_24902_MOESM1_ESM.docx]

**SUPPLEMENTARY INFORMATION**

**Electrochemical affinity biosensors for fast detection of gene-specific methylations with no need for bisulfite and amplification treatments**

Eloy Povedano,^1^ Eva Vargas,^1^ Víctor Ruiz-Valdepeñas Montiel,^1^ Rebeca M. Torrente-Rodríguez,^1^ María Pedrero,^1^ Rodrigo Barderas,^2^ Pablo San Segundo-Acosta,^2^ Alberto Peláez-García,^3^ Marta Mendiola,^4^ David Hardisson,^3,5^ Susana Campuzano,^1,*^ José M. Pingarrón^1,*^

^1^Departamento de Química Analítica, Facultad de CC. Químicas, Universidad Complutense de Madrid, E-28040 Madrid (Spain).

^2^Unidad Funcional de Investigación de Enfermedades Crónicas, Instituto de Salud Carlos III, 28220 Majadahonda, Madrid (Spain).

^3^Department of Pathology, Molecular Pathology and Therapeutic Targets Group, Hospital Universitario La Paz IdiPAZ, Madrid (Spain).

^4^Molecular Pathology and Therapeutic Targets Group and Molecular Pathology Section, INGEMM, Hospital Universitario La Paz IdiPAZ, Madrid (Spain).

^5^Facultad de Medicina, Universidad Autonoma de Madrid, Madrid (Spain).

**Figure S1 ⏐** **Optimization of experimental variables.** Influence of the capture (a), detector (b) antibodies and target DNA (c) incubation times used in the immunosensor fabrication, and the bCp (d), target DNA (e) incubation times, detector (f), HRP-secondary (g) antibodies concentration and mixture antibodies incubation time (h) used in the preparation of the DNA sensor, on the amperometric responses measured with the developed biosensors for 0.0 (white bars) and 5.0 nM of synthetic target *RASSF1A* (grey bars) and the corresponding S/B ratio values (in red). Error bars estimated as triple of the standard deviation of three replicates.
